# To Help or Not to Help: Intervening in Cyberbullying Among Chinese Cyber-Bystanders

**DOI:** 10.3389/fpsyg.2021.483250

**Published:** 2021-07-14

**Authors:** Angel Nga Man Leung

**Affiliations:** Department of Psychology, The Education University of Hong Kong, Hong Kong, China

**Keywords:** cyberbullying, cyber-bystanders, intervening behavior, Chinese college students, intention

## Abstract

Cyberbullying has become a serious concern among Internet users worldwide. However, relatively little is known about individuals who witness cyberbullying and how they behave. A bystander is someone who sees bullying or other forms of aggressive or violent behavior that targets someone else and who may choose to respond by either being part of the problem (a hurtful bystander), or part of the solution (a helpful bystander). Few studies examined the phenomena of cyber-bystanders in Chinese populations. Guided by the five-step bystander theoretical model and the theory of planned behavior, this study, addressed this gap to understand how the characteristics of cyber-bystanders explained their intervention in cyberbullying in a Chinese population. This study tested two preregistered hypotheses: (1) controlling for age and gender, awareness of cyberbullying, attitudes, subjective norm and perceived behavioral control to intervene; plus past experience with cyberbullying (measured as past experience in cyberbullying perpetration and victimization), felt responsibility, and self-efficacy to intervene with regard to cyberbullying would explain the intention of cyber-bystanders to intervene in cyberbullying, and (2) the intention of cyber-bystanders to intervene cyberbullying would positively explain their intervening behavior. A total of 581 college students with experience of witnessing cyberbullying were included in the analysis. Applying structural equation modeling with observed variables, a path analysis model was built to test the hypotheses; this study also conducted exploratory analyses by including direct paths from the characteristics of cyber-bystanders to explain intervening behavior. Results found that only awareness of cyberbullying, a subjective norm, and self-efficacy to intervene positively explained intention to intervene cyberbullying; therefore, hypothesis 1 was partly supported. Also, intention to intervene cyberbullying positively explained intervening behavior; therefore, hypothesis 2 was supported. For the exploratory analysis, intention to intervene partially mediated the relation between a subjective norm to intervene and intervening behavior; and intention to intervene also partially mediated the relation between self-efficacy to intervene and intervening behavior. In addition, past experience in cyberbullying victimization also positively and directly predicted intervening behavior. Findings provided a foundation for designing future intervention programs to mobilize cyber-bystanders to become “upstanders.”

Cyberbullying is defined as “long-term, aggressive, intentional, and repetitive acts by one or more individuals using electronic means against an almost powerless victim” (Dehue, 2013, p. 2). While being bullied in traditional physical settings increased the risks of both internalized and externalized problems (e.g., Prino et al., 2019), being cyberbullied also poses significant psychological threats to adolescents, which include increased depressive symptoms, poor academic performance, loneliness, as well as other socio-emotional problems (Olenik-Shemesh et al., 2012; Schenk and Fremouw, 2012; Wigderson and Lynch, 2013; Na et al., 2015; Tennant et al., 2015). In traditional school bullying, students can take up a role in the bullying event, such as bullies, victims, bullied victims (Marengo et al., 2018), or bystanders (Longobardi et al., 2019). Similarly, cyberbullying also involves a number of cyber-bystanders. However, research on cyber-bystanders is limited. Unlike in traditional face-to-face bullying, a single cyberbullying incident can “snowball” and go viral online because of the potentially unlimited number of individuals who are online and witness the cyberbullying incidents. These individuals who witness cyberbullying may then become cyber-bystanders who share, comment, or forward the details of cyberbullying incidents to countless others (Slonje et al., 2013), resulting in widespread humiliation and victimization. Cyber-bystanders may reinforce the frequency of cyberbullying because their presence or responses may fulfill the agentic goals of cyberbullies of being admired, feeling dominant, and powerful (Salmivalli, 2010). On the other hand, some cyber-bystanders may choose to stop cyberbullying by calling attention to the incident, helping or defending the victims, or stopping to share or comment on cyberbullying incidents. Bystanders who witness bullying, in both traditional physical and online contexts, make up a large proportion of those who are involved in bullying incidents of any sort. Studies found that 23–85% of students have reported being involved as bystanders in traditional bullying situations (e.g., Pepler and Craig, 1995; Quirk and Campbell, 2015), while 10–91% reported being involved as cyber-bystanders (e.g., Lenhart et al., 2011; Quirk and Campbell, 2015). Schultze-Krumbholz et al. (2018) also found that among adolescents who were involved in cyberbullying, cyber-bystanders (who defended the victims or remained as outsiders) made up the largest group. Therefore, cyber-bystanders who intervene the incidents may play key roles in developing, maintaining, or stopping of the “vicious cycle” of cyberbullying.

Building on two classic models, the *five-step bystander intervention model* (Latané and Darley, 1970) and the *theory of planned behavior* (TPB; Ajzen, 1991), along with an integrating model suggested by Desmet et al. (2014); DeSmet et al. (2016), this study examined the socio-cognitive factors that explain the intention and the likelihood of intervening in cyberbullying incidents. Cyber-bystander behaviors in a Chinese population in Hong Kong, China, were examined for two reasons: (1) behaviors of bystanders may be influenced by cultural or societal values (e.g., Pozzoli et al., 2012); however, most studies on cyber-bystanders were carried out in Western settings (e.g., Desmet et al., 2014; DeSmet et al., 2016); and (2) currently, cyberbullying is a criminal offense in the United Kingdom, United States, Australia, New Zealand, yet there is no legislation against cyberbullying in Hong Kong; and cyberbullying is rarely discussed or addressed in the curricula of most Hong Kong local schools or colleges. Therefore, the experience of the responses to and the interpretation about cyberbullying of Chinese students in Hong Kong, China could be different from the patterns reported for their Western counterparts.

In addition, Schwartz et al. (2001) suggested that being sensitive to others and minimizing interpersonal conflicts are highly valued in the Chinese culture. Therefore, self-control and interdependent self-construal are cultivated and socialized. For instance, the socialization process such as a parenting style called “guan,” is commonly observed in the Chinese population but not their western counterparts (e.g., Lan et al., 2019). Moreover, timid behavior or seemingly shy behavior could be a reflection of the cultural emphasis on self-restraint and behavioral inhibition rather than being unable to protect oneself (Xu and Farver, 2009). Therefore, there may be cultural differences in the belief systems of students and their attitudes toward cyber-bullying and cyber-bystanding behaviors, as well as in their perceived norm as outlined in the theory of planned behavior. Moreover, as suggested by Romera et al. (2017), most research on cyberbullying has been conducted primarily in North America and Europe, and the importance of culture has been overlooked. Indeed, studies have suggested that Chinese students tended to report cyberbullying incidents to adults, a response that may be influenced by Confucian beliefs (Li, 2008).

Currently, there are few studies of cyber-bystanders in Chinese populations. Huang and Chou (2010) reported gender differences (with females reporting fewer cyber-bystanding experiences), and cyber-bystanders were more likely to become cyber-victims as compared with cyber-bullies. Zhou et al. (2018) found that males reported more by-standing behaviors, and that moral disengagement partially mediated the relation between neuroticism and bystander behavior. Li et al. (2013) found that almost 90% of Chinese school students had been cyber-bystanders, and there were significant positive correlations among being a cyberbully, a cyber-victim, and cyber-bystander. Mojdehi et al. (2019) compared the perspectives of Chinese, Persian, and Canadian youths on cyberbullying events as cyber-bystanders. They found that Persian youth evaluated cyberbullying less negatively than Canadian and Chinese youth; while Canadian and Chinese youth rated the behaviors of perpetrators more negatively than their Persian counterparts. To the best of the knowledge of the author, studies that have comprehensively examined the belief system or socio-cognitive factors that predict the behavior of cyber-bystanders among Chinese students are scarce. This study, therefore, addressed this gap and allowed us to understand how the characteristics of cyber-bystanders explain their intervention in cyberbullying in a Chinese population.

The five-step bystander intervention model (Latané and Darley, 1970) has been well-validated to examine behaviors of bystanders in various social situations (e.g., Pozzoli and Gini, 2013; Nickerson et al., 2014). For bystanders to intervene, they must: (1) notice that an event is taking place; (2) interpret the incident as an emergency or requiring an action of some kind; (3) feel a responsibility to take action; (4) know how to apply for the appropriate assistance; and (5) take action or choose to help. The current study applied this model to the online context.

Noticing cyberbullying is the first component in the five-step bystander intervention model. For individuals to notice and interpret an event as an emergency, the event has to be vivid (Dovidio et al., 2006; Loewenstein and Small, 2007). Because cyber-bystanders cannot observe the emotional responses of the cyber-victims, they may underestimate the severity of the situation (Heirman and Walrave, 2008), which may result in fewer intervening behaviors. In addition, most adolescents tend to interpret cyberbullying as something that is for fun, and only about 50% are aware of cyberbullying (Runions et al., 2013). Furthermore, bullying in the online context tends to be ambiguous and thus poses difficulties for cyber-bystanders to notice or interpret an event as cyberbullying (Bastiaensens et al., 2014; Van Cleemput et al., 2014). Past studies suggested that being aware of the consequences of cyberbullying and knowing how to act pro-socially promotes healthy online behavior in adolescents (Cowie and Colliety, 2010). Dillon and Bushman (2015) found that students who noticed cyberbullying were more likely to intervene. Nickerson et al. (2014) also confirmed that noticing harmful events is essential for bystander intervention to take place. Greitemeyer et al. (2006) found that the speed of noticing aggressive events predicted helping behaviors. Therefore, the first aim of this study was to understand how awareness of cyberbullying is related to the intervening behavior of cyber-bystanders among Chinese students.

Interpreting cyberbullying is the second component in the five-step bystander intervention model. How individuals interpret an event as cyberbullying depends on their belief systems. According to the theory of planned behavior (TPB; Ajzen, 1991), belief systems include attitudes (A), subjective norm (SN), and perceived behavioral control (PBC), and intention to behave. Attitudes are general affective evaluations of an individual of the behavior of another. Subjective norm involves the beliefs of individuals about how others they care about view or approve their behavior. Perceived controlled behavior refers to the perceived difficulty/self-efficacy of individuals in responding or carrying out an action. These three elements (i.e., attitudes, subjective norm, and perceived behavioral control) can predict the intention of individuals to act and their actual behavior. Few studies on cyberbullying demonstrated that certain elements of TPB (e.g., attitudes; Pornari and Wood, 2010; and subjective norm; Wright and Li, 2013) can be applied to explain cyberbullying. Heirman and Walrave (2012) found that elements of A, SN, PCB, and intention to behave explained some of the variances in the perpetration of cyberbullying. Nevertheless, only a few studies have applied the attitude component of TPB in predicting behaviors of cyber-bystanders, with two exceptions. Work by Pabian et al. (2016) suggested that positive attitudes toward cyberbullying (i.e., accepting cyberbullying) predict later bystander behaviors. DeSmet et al. (2016) proposed an integrative model based on TPB and environmental influences. The results showed that the attitudes of students toward cyber-bystanding predicted their cyber-bystanding behaviors. Still, no studies examined the belief system of cyber-bystanders in a Chinese population. Therefore, the second aim of this study was to investigate how the belief system of cyber-bystanders (i.e., A, SN, and PBC) explained the intervening behavior of cyber-bystanders.

*Felt responsibility to intervene cyberbullying* is the third component in the five-step bystander intervention model. Due to the large number of cyber-bystanders in a typical online context, felt responsibilities of individuals to intervene cyberbullying are often diffused. Moreover, attributions of cyber-bystanders attributions about victim characteristics further diffuse their intentions and felt responsibilities to intervene. According to the attribution theory of Weiner (1986), if bystanders perceive that the victims of bullying are responsible for the bullying or that these victims should be blamed, the bystanders may be less likely to offer help. Van Cleemput et al. (2014) found that, when cyber-bystanders believed that it should be the responsibility of the victims to act, the cyber-bystanders would not intervene, because if the victims provoked the bullies first, the cyber-bystanders tended to think that the victims were “deserved” to be bullied. In addition, if the cyber-bystanders perceived that the victims were their friends, they were then more likely to feel responsible to intervene. Obermaier et al. (2016) reported that felt responsibility of cyber-bystanders mediated the relation between a number of bystanders and intention to intervene. The third aim of this study, therefore, was to examine the relationship between the felt responsibility of cyber-bystanders to intervene and their likelihood of intervening.

Finally, *knowing how to intervene* in the five-step bystander intervention model determines whether individuals take action to intervene in a cyberbullying incident. In traditional physical bullying settings, the lack of appropriate intervention skills is predictive to the non-intervening behavior of the bystanders (Burn, 2009). Similarly, in the online context, cyber-bystanders may not have enough information and communication technology (ICT) knowledge to intervene or report the incidents. Self-efficacy refers to the confidence of an individual to accomplish a specific task (Bandura et al., 1996) and, in this context, the ability to defend cyberbullying victims. Desmet et al. (2014); DeSmet et al. (2016) found that self-efficacy encouraged positive upstanding behavior by influencing intention to intervene in cyberbullying events. Therefore, the last aim of this study was to investigate whether self-efficacy in intervening cyberbullying predicts cyberbullying.

Past research has shown high prevalence rates of cyberbullying among undergraduate students (e.g., Dilmaç, 2009; Minor et al., 2013; Faucher et al., 2014). In the United States, between 4.3 and 21% of college students reported have been bullied online (Finn, 2004; MacDonald and Roberts-Pittman, 2010; Webber and Ovedovitz, 2018). About 36% of college students in Spain suffered from being disseminated with lies and rumors online (Yubero et al., 2017). Another study showed that, in Greece, 58.4% of college students participated in cyber-bullying incidents (Kokkinos et al., 2014). In Hong Kong, Leung et al. (2018b) found that 58% of college students reported cyberbullying others, and 68% reported being cyber-victimized. Although the prevalence rate of cyberbullying among college students is not low, few studies have targeted this age group. Therefore, this study examined the mechanism of cyberbullying intervention in a sample of Hong Kong Chinese college students.

This study also explored the relevance of prior cyberbullying involvement to the belief system and intervening behaviors of an individual to cyberbullying incidents. For instance, past studies suggested that experience with cyberbullying or being cyberbullied predicted perceived behavioral control and subjective norm toward cyberbullying (e.g., Heirman and Walrave, 2012). Concerning the intervening behavior of cyber-bystanders, Van Cleemput et al. (2014) found that victims of cyberbullying were more likely to demonstrate positive intervening behavior when they witnessed cyberbullying. Desmet et al. (2014) also found that past experience with being cyberbullied positively predicted helping behavior among adolescents. Therefore, the prior involvement of students in cyberbullying was included in the present model as a predictor of intervening behavior.

Finally, evidence for the effect of gender and age on the behavior of cyber-bystanders has been mixed. Barlińska et al. (2013) and Machackova et al. (2013) found that gender and age did not significantly predict helping behavior of bystanders, whereas DeSmet et al. (2016) found that girls were more likely to demonstrate positive intervening behavior. Other studies conducted among young adolescents found that, with increasing age, they were less likely to help victims when witnessing cyberbullying (e.g., Van Cleemput et al., 2014; Erreygers et al., 2016). As the predictive power of gender and age remains unclear, and few studies have focused on college populations, gender and age were added as controlled variables (i.e., covariates) in the current study.

The primary goal was to understand how characteristics of cyber-bystanders, namely their awareness of cyberbullying, belief systems of cyber-bystanders (i.e., attitudes, subjective norm, and perceived behavioral control to intervene), past involvement in cyberbullying, felt responsibility, and self-efficacy with regard to cyberbullying intervention predict intention of individuals to intervene in cyberbullying.

Figure 1 displays the preregistered conceptual model. There were two hypotheses:

Hypothesis 1: Controlling for gender and age, awareness of cyberbullying, attitudes, subjective norm, and perceived behavioral control to intervene, plus felt responsibility, past involvement in cyberbullying (measured as past experience in cyberbullying perpetration and victimization), and self-efficacy with regard to intervention would explain the intention of cyber-bystanders to intervene cyberbullying.
Hypothesis 2: Controlling for gender and age, the intention of cyber-bystanders to intervene cyberbullying would positively explain their intervening behavior and the likelihood of defending the victim.

**Figure 1:**
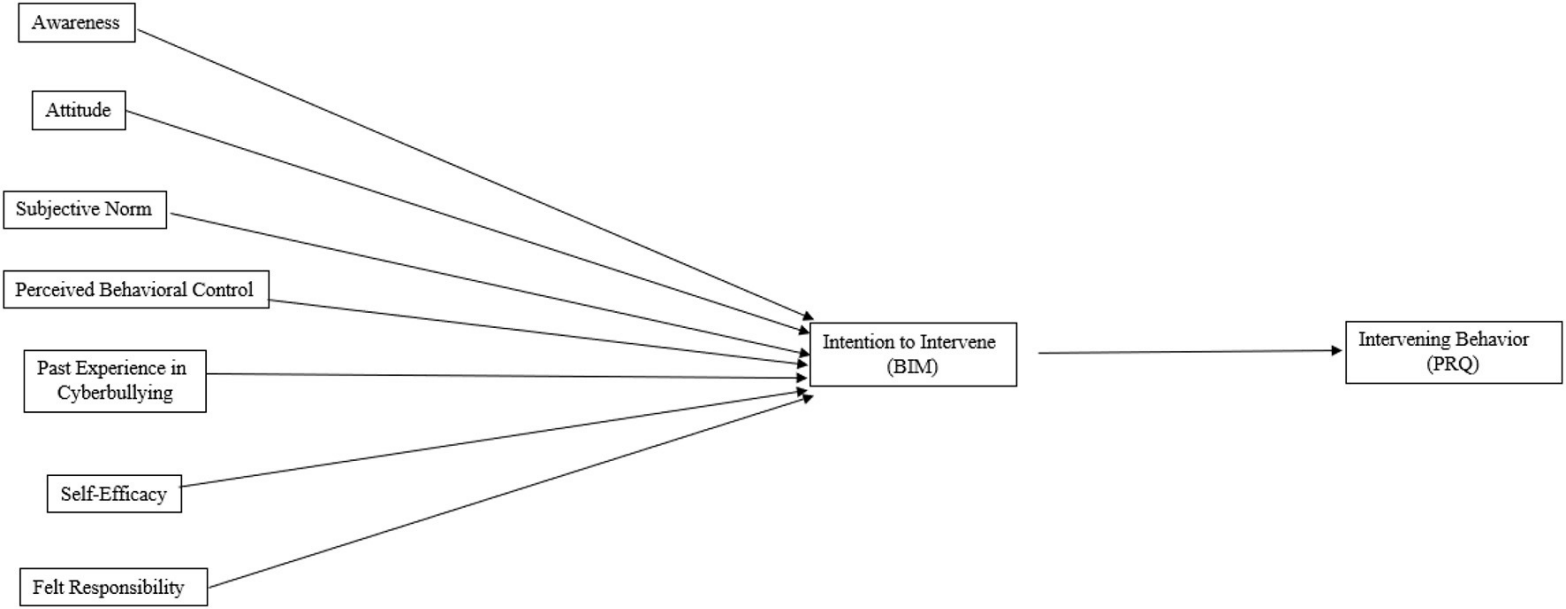
Proposed conceptual model of the preregistered hypotheses. For simplicity, controlled variables (age and gender) and concurrent relations among predictors are not shown in the conceptual model. Past experience in cyberbullying was measured as “experience in cyberbullying victimization” and “experience in cyberbullying victimization” separately.

## Method

### Participants

A total of 699 college students aged below 30 years old answered a 10-item scale adopted from Leung et al. (2018c) to measure the frequency of witnessing cyberbullying in the past 3 months. The scale was used with Hong Kong Chinese students in a prior study to measure cyberbullying involvement, and it was adopted to measure if the participants witnessed such behavior online. A sample item is “I witness others gossip or say mean things about other students on the Internet,” on a scale from “never” = 1, to “always” = 5. The composite score was created by adding up the scores of 10 items; individuals who never witnessed any cyberbullying would have a composite score from this scale.

As this study aimed at understanding the behavior of cyber-bystanders, only 581 participants (*M* = 20.46, *SD* = 1.78; males = 134, females = 447) who had past experience as cyber-bystanders (i.e., had a composite score >10) were included in the subsequent analysis; in other words, 83.1% of college students in the current study witnessed cyberbullying, which was similar to past studies. For instance, Lenhart et al. (2011) found that 88% of students had witnessed cyberbullying; a study in Hong Kong also showed that about 90% of students in Hong Kong witnessed cyberbullying (Leung, 2018).

### Measures

#### Basic Demographics

Age, gender, year of study, and time spent online were measured.

#### Past Experience in Cyberbullying

Similar to bullying in the physical context, researchers suggested that clarifications on the definition and measurement of bullying are needed (e.g., Volk et al., 2017). Although aggression and bullying overlap, they are not identical, particularly in terms of power differences and being repetitive, which are signatures of bullying but not necessarily of aggression (Hawley et al., 2011). In the online context, nevertheless, because of its anonymous nature, it could be difficult to detect the power difference between the bullies and the victims. Therefore, as suggested by Volk et al. (2017), when there was “no gold standard measure of bullying” (p. 41), the most suitable measurements should be chosen to test the hypotheses. Among the few studies that offered a clear definition of cyberbullying, Langos (2012) suggested the clear and concise definitions of cyberbullying should be:

Cyberbullying involves the use of ICTs to carry out a series of acts as in the case of direct cyberbullying, or an act as in the case of indirect cyberbullying, intended to harm another (the victim) who cannot easily defend himself or herself. Direct cyberbullying involves a perpetrator repeatedly directing unwanted electronic communications to a victim who cannot easily defend himself or herself with the intent to harm the victim. Indirect cyberbullying involves directing a single or repeated unwanted electronic communications to a victim who cannot easily defend himself or herself with the intent to harm the victim. An intention to harm is established where a reasonable person, adopting the position of the victim and having regard to all the circumstances, would regard the series of acts or an act as acts or an act intended to harm the victim (p. 288).

The participants were given the aforementioned definition of cyberbullying, and they indicated how frequently they have been involved in various kinds of behaviors that constitute cyberbullying (as perpetrators or victims), using the nine-item cyberbullying and cyber-victimization scales by Patchin and Hinduja (2015). A sample item for the cyberbullying perpetration scale is “I cyberbullied others”; Cronbach's alpha = 0.96. A sample item for the cyber-victimization scale is “I have been cyberbullied”; Cronbach's alpha = 0.95. The two scales were developed by Patchin and Hinduja (2015), two renowned researchers in the field of cyberbullying. Both scales demonstrated strong initial validity and reliability in 10 different surveys, which involved more than 15,000 students.

#### Awareness of Cyberbullying

The participants rated six items on a seven-point scale (1 = strongly disagree; 7 = strongly agree) to measure their cyberbullying awareness (Brewer, 2011). A sample item is: “People are negatively affected by cyberbullying.” This scale was used by Leung et al. (2018a) with a Hong Kong Chinese sample, Cronbach's alpha = 0.80. Cronbach's alpha of this scale in the present study was 0.75.

#### Attitudes Toward Cyberbullying

The attitudes toward cyberbullying questionnaire (PACQ; Barlett and Gentile, 2012) consists of nine items. The participants rated on a seven-point scale (1 = strongly disagree; 7 = strongly agree). This scale was used in a Hong Kong Chinese sample before, with a Cronbach's alpha of 0.86 (Leung et al., 2018a). A sample item is, “Sometimes using passive aggressive methods of sending mean e-mails to others is the only way to get even.” The scale was reversed code so that a high score means a more negative attitude toward cyberbullying (i.e., believing that cyberbullying is not good). Cronbach's alpha of this scale in the present study was 0.86.

#### Subjective Norm (SN)

The participants rated four questions adapted from Kraft et al. (2005), using a seven-point scale (1 = strongly disagree; 7 = strongly agree) to measure their subjective norms about bystander behavior. A sample item is: “Most people who are important to me would like me to intervene in a cyberbullying incident.” Cronbach's alpha of this scale in the present study was 0.88.

#### Perceived Behavioral Control (PBC) on Intervening Behavior

The participants completed a nine-item scale adapted from Kraft et al. (2005) to measure their perceived behavioral control, using a seven-point scale (1 = strongly disagree; 7 = strongly agree). The items were adjusted to fit into the online context. For example, “I have full control over my intervening behavior when I witness cyberbullying incidents.” Cronbach's alpha of this scale in the present study was 0.84.

#### Intention to Intervene in a Cyberbullying Context

The participants completed the 12-item bystander intervention measure (Koon, 2013; reliability > 0.70), using a five-point scale (1 = strongly unlikely to intervene; 5 = strongly likely to intervene). A sample item is “Privately advise the victim to block the harasser.” Cronbach's alpha of this scale in the present study was 0.87.

#### Felt Responsibility to Intervene

The participants rated a three-item scale to measure their felt responsibility to intervene in a cyberbullying situation, using a five-point Likert (1 = strongly disagree; 5 = strongly agree; Obermaier et al., 2016). A sample item is “I highly feel personally responsible to support the cyber-victim.” Cronbach's alpha of this scale in the present study was 0.84.

#### Self-Efficacy to Intervene Cyberbullying

The participants rated their self-efficacy to intervene in cyberbullying, using 10 items adopted from Schwarzer and Jerusalem (1995) on a seven-point scale (1 = strongly disagree; 7 = strongly agree); e.g., “I have confidence that I can effectively resolve urgent cases of cyberbullying.” Modifications were made to fit the cyberbullying context. It was used in the Hong Kong Chinese sample, with Cronbach's alpha = 0.96 (Leung et al., 2019). Cronbach's alpha of this scale in the present study was 0.93.

#### Intervening Behavior

The participants completed a three-item scale adopted from the participant role questionnaire (Salmivalli and Voeten, 2004). This scale measures the frequency of bystanders to intervene when witnessing aggression and the likelihood to defend the victim, using a five-point Likert scale, from “1” as never to “5” as always. The items will be adjusted to fit in the cyberbullying context. A sample item is, “Tell others to stop cyberbullying.” Cronbach's alpha of this scale in the present study was 0.87.

### Procedure

Local Hong Kong college students were invited via mass emails on campus. They were given a link to access the online questionnaire. Ethics approval was obtained from the University of the author. A consent form was shown on the first page of the questionnaire. The participants were given a HK$50 coupon (~USD$6) for their participation.

## Results

Table 1 shows the descriptive statistics of the demographic variables. Table 2 shows the descriptive statistics, internal reliabilities, and correlations of the measured study variables. Table 3 shows the gender differences in our study variables, using independent samples *t*-test analyses.

**Table 1:** Descriptive statistics of demographic variables.

**Variable**	**Mean (SD)/*N* (%)**
Age	20.46 (1.78)
**Gender**	
Males	134 (23.1)
Females	447 (76.9)
Time spent online (hours)	2.82(2.60)

**Table 2:** Descriptive statistics, internal reliability, and correlations of study variables.

**Variable**	**Mean (SD)**	**Cronbach alpha**	**Awareness**	**Attitude**	**Subjective norm**	**Perceived behavioral control**	**Past experience in cyberbullying perpetration**	**Past experience in cyberbullying victimization**	**Felt responsibility**	**Self-efficacy**	**Intention to intervene**	**Intervening behavior**
Awareness	32.02 (5.16)	0.75	1									
Attitude	50.75 (8.21)	0.86	0.361^**^	1								
Subjective norm	16.50 (4.45)	0.88	0.136^**^	0.022	1							
Perceived behavioral control	31.92 (7.74)	0.85	0.003	−0.027	0.408^**^	1						
Past experience in cyberbullying perpetration	0.90 (3.35)	0.96	−0.262^**^	−0.410^**^	0.008	0.084^*^	1					
Past experience in cyberbullying victimization	1.34 (4.00)	0.95	−0.252^**^	−0.370^**^	0.043	0.087^*^	0.809^**^	1				
Felt responsibility	8.71 (2.62)	0.84	0.101^*^	0.072	0.406^**^	0.255^**^	−0.027	0.020	1			
Self-efficacy	34.93 (10.05)	0.93	0.058	0.015	0.436^**^	0.597^**^	0.037	0.055	0.338^**^	1		
Intention to intervene	46.73 (11.42)	0.87	0.136^**^	−0.013	0.365^**^	0.341^**^	0.004	−0.015	0.260^**^	0.411^**^	1	
Intervening behavior	4.99 (2.26)	0.87	0.049	−0.074	0.274^**^	0.226^**^	0.209^**^	0.241^**^	0.206^**^	0.303^**^	0.291^**^	1

* *p < 0.05*,

** *p < 0.001*.

**Table 3:** Independent samples *t*-test comparing gender differences in study variables.

**Variable**	**Gender**	***N***	***M***	***SD***	***t***	***p***
Awareness	Male Female	134 447	29.46 32.79	5.92 4.64	−5.98	<0.001
Attitude	Male Female	134 447	45.80 52.23	8.89 7.38	−7.63	<0.001
Subjective norm	Male Female	134 447	16.76 16.43	4.35 4.48	0.76	0.446
Perceived behavioral control	Male Female	134 447	33.31 31.50	7.10 7.89	2.39	0.017
Past experience in cyberbullying perpetration	Male Female	134 447	2.47 0.43	5.80 1.91	4.02	<0.001
Past experience in cyberbullying victimization	Male Female	134 447	3.23 0.77	6.51 2.60	4.27	<0.001
Felt responsibility	Male Female	134 447	8.66 8.73	2.52 2.66	−0.28	0.779
Self-efficacy	Male Female	134 447	35.81 34.66	9.66 10.16	1.15	0.249
Intention to intervene	Male Female	134 447	47.24 46.57	11.05 11.54	0.59	0.554
Intervening behavior	Male Female	134 447	5.32 4.86	2.36 2.20	2.10	0.036

Results of the correlational analysis showed that awareness of cyberbullying, subjective norm, perceived behavioral control to intervene, felt responsibility, and self-efficacy with regard to the intervention were positively correlated with intention to intervene cyberbullying; while the intention to intervene was positively correlated with intervening behavior (see Table 2).

To test hypotheses 1 and 2, the relations among the measured variables were further investigated, using path analysis (a form of SEM with observed variables), using lavvan package (Rosseel, 2012) in R (R Development Core Team, 2012); age and gender were included in the model as covariates in explaining both intention to intervene and intervening behavior. An exploratory analysis was conducted by including direct paths from characteristics of cyber-bystanders to intervening behavior as well.

**Figure 2:**
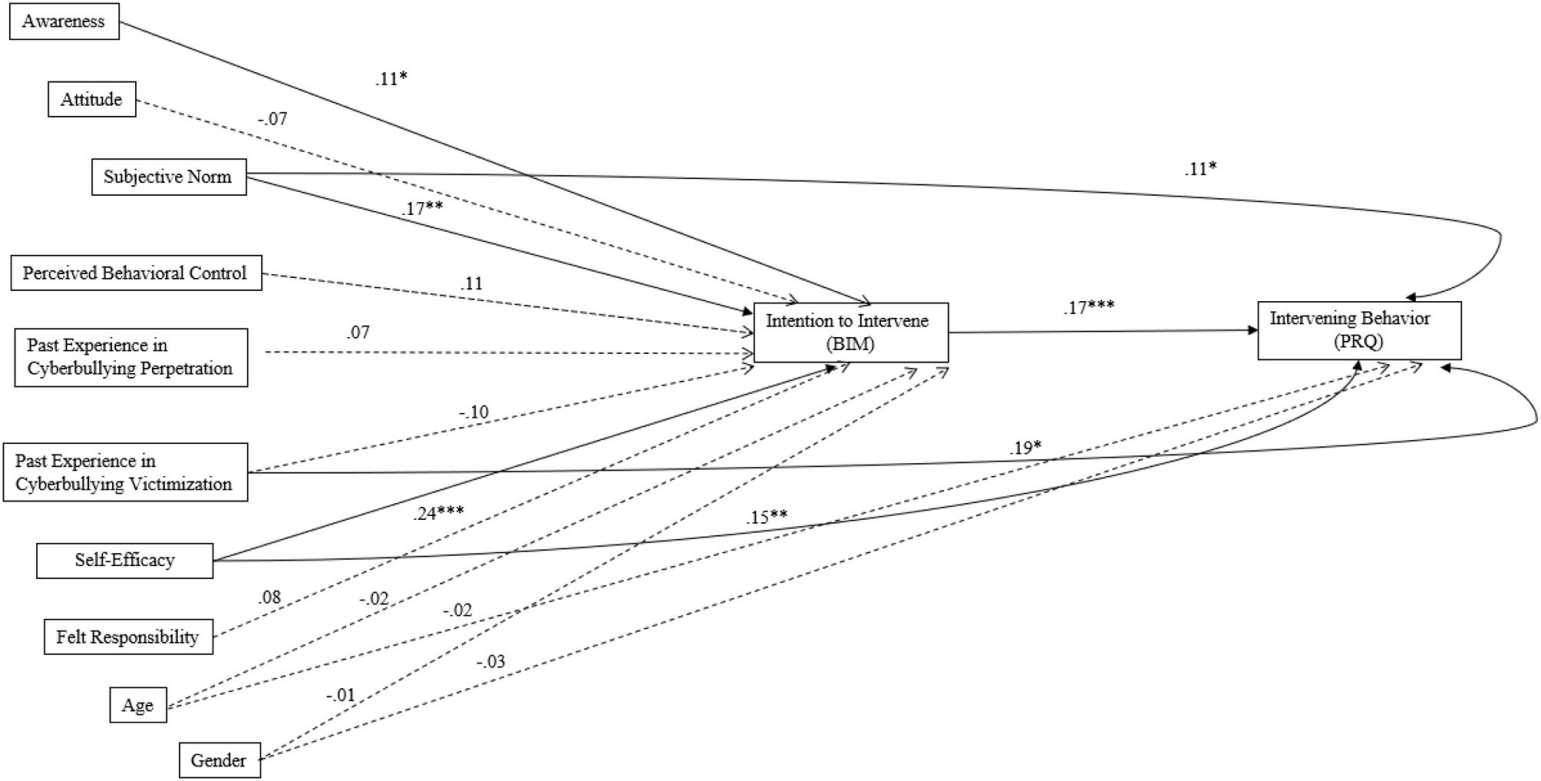
Results of path analysis among variables. Values in path analysis represent standardized regression coefficients. Solid lines and dotted lines represent significant paths and non-significant paths, respectively. *p < 0.05, **p < 0.01,***p < 0.001.

As indicated in Figure 2, for hypotheses 1 and 2, when all the measured characteristics of cyber-bystanders were included in the model of path analysis, only awareness of cyberbullying (beta = 0.11, *p* = 0.018), subjective norm (beta = 0.17, *p* = 0.002), and self-efficacy (beta = 0.24, *p* < 0.001) to intervene positively and significantly explained intention to intervene. Also, intention to intervene (beta = 0.17, *p* < 0.001) positively explained intervening behavior.

For the exploratory analysis, results showed that subjective norm (beta = 0.10, *p* = 0.011) and self-efficacy to intervene (beta = 0.15, *p* = 0.003) positively and directly explained intervening behavior of cyber-bystanders; while past experience in cyberbullying victimization (beta =0.19, p = 0.033) positively and directly explained the intervening behavior, despite of the fact that it did not explain intention to intervene. Other direct paths from awareness (beta = 0.07, p = 0.125), attitude (beta = −0.003, p = 0.948), perceived behavioral control (beta = −0.01, p = 0.893), past experience in cyberbullying perpetration (beta = 0.06, p = 0.493), and felt responsibility to intervening behavior (beta = 0.06, p = 0.134) were not significant. As analysis of direct paths to intervening behavior was added as exploratory analysis, for the sake of simplicity, only significant direct paths are included in Figure 2. Characteristics of cyber-bystanders added in the model accounted for 23.8% in the total variance of the intention of cyber-bystanders to intervene in cyberbullying, while all these characteristics, along with intention to intervene cyberbullying, accounted for 20.0% of variance in intervening behavior.

The indirect effect was tested, using a percentile bootstrap estimation approach, with 1,000 samples. Results showed that subjective norm had significant direct effect (B = 0.05, SE = 0.02, 95% CI [0.01, 0.09], beta = 0.10, *p* = 0.011), indirect effect *via* intention to intervene (B = 0.02. SE = 0.01, 95% CI [0.01, 0.03], beta = 0.03, *p* = 0.011), and total effect (B = 0.07, SE = 02, 95% CI [0.03, 0.11], beta = 0.13, *p* = 0.001) on intervening behaviors. Results also showed that self-efficacy had the significant direct effect (B = 0.03, SE = 0.01, 95% CI [0.01, 0.06], beta = 0.15, *p* = 0.003), indirect effect *via* intention to intervene (B = 0.01, SE = 0.003, 95% CI [0.004, 0.02], beta = 0.04, *p* = 0.002), and total effect (B = 0.04, SE = 0.01, 95% CI [0.02, 0.06], beta = 0.19, *p* < 0.001) on intervening behaviors. In other words, intention to intervene cyberbullying partially mediated the relations between subjective norm and self-efficacy to intervene, respectively, to intervening behavior of cyber-bystanders.

## Discussion

Guided by the two classic models, the five-step bystander intervention model (Latané and Darley, 1970) and the theory of planned behavior (TPB; Ajzen, 1991), along with an integrating model suggested by Desmet et al. (2014); DeSmet et al. (2016), this study aimed at studying a bundle of characteristics of cyber-bystanders together and to test if they explained the intention of cyber-bystanders to intervene and their intervening behavior. It was among the first few studies to investigate the underlying socio-cognitive mechanism of intervening behavior of cyber-bystanders among Chinese students, a population that has been under-researched in the existing literature. In the path analysis model, controlling for gender and age, awareness of cyberbullying, subjective norm, and self-efficacy to intervene positively and significantly explained intention to intervene cyberbullying. Therefore, hypothesis 1 was partially supported. Results of the path analysis model also showed that intention to intervene positively and significantly explained intervening behavior; therefore, hypothesis 2 was supported. Exploratory analysis of direct paths from characteristics of cyber-bystanders to intervening behavior further suggested that past experience in cyber-victimization positively and directly explained intervening behavior; while the intention to intervene partially mediated subjective norm and self-efficacy to intervene to intervening behavior, respectively.

According to the five-step intervention model, noticing or being aware of an emergency is the very first step for any intervention to take place (Latané and Darley, 1970), while the second step involves interpretation of individuals of the event, and this is affected by belief systems of individuals, which can further be explained by TPB, which includes attitudes (A), subjective norm (SN), and perceived behavioral control (PBC). The third and fourth steps to intervene involve felt responsibility and self-efficacy to intervene, while the last step is the intervening behavior. As TPB suggested that intention to engage in a behavior predicts the behavioral responses of individuals, intention to intervene cyberbullying was included as a mediator in the current study.

Awareness of cyberbullying is the first step of the five-step intervention model. Consistent with past literature, which suggested the importance of noticing cyberbullying (e.g., Dillon and Bushman, 2015), this study supported that, among Chinese college students who witnessed cyberbullying, their awareness of cyberbullying positively explained their intention to intervene, which, in turn, positively explained their intervening behavior. To prevent bullying in school settings, the first step in the bullying intervention program in the face-to-face context was to raise awareness of students (e.g., Salmivalli, 1999; Salmivalli et al., 2005). Findings of the present study provided empirical evidence that such awareness is also important when bullying happened in the online context.

Nevertheless, few interventions in existing literature have targeted this age group, and only a handful of studies were conducted to increase awareness or knowledge of college students of cyberbullying (e.g., Doane et al., 2016). Therefore, the future intervention program in tertiary education should consider raising awareness of cyberbullying of college students. A potentially effective way to increase awareness of cyberbullying of college students is to adopt an experiential learning approach. For instance, Leung et al. (2018a) conducted one of the very few studies that aimed at addressing cyberbullying among Hong Kong Chinese college students. In the 1-h short intervention program, college students actively participated in a Facebook role play activity, watched a documentary about cyberbullying, and involved in a discussion and a self-reflection writing task. With an experimental learning design, students experienced and understood the feelings of the cyber-victims; their sympathy for victims was boosted, and their cognitive experience was challenged *via* group discussion. Leung et al.'s (2018a) study showed that (a) the participants who were in the intervention group increased awareness of cyberbullying, as compared with the control group; and (b) among the participants who reported themselves being highly engaged in the intervention, such effect was maintained in an 8-week follow-up. Therefore, it is suggested that similar intervention programs that promote awareness of college students of cyberbullying should be included in the tertiary education curriculum in the future.

The second step of the five-step intervention model involves how individuals interpret an event; such a process is likely to be affected by how individuals perceive their important referents want them to conduct a behavior. Results of this study found that the higher the subjective norm that participants believed their important referents would approve them to intervene cyberbullying, the higher their intention to intervene cyberbullying was, which, in turn, positively explained intervening behavior. This is consistent with past studies on bullying in face-to-face context. Past studies reported that if students believed that their parents and friends expected them to support victims of bullying, they expressed higher intention to intervene (e.g., Rigby and Johnson, 2006). In the online context, Bastiaensens et al. (2016) found that among those who witnessed cyberbullying, when they thought that their peers would approve of cyberbullying behavior, they would experience more social pressure to join in the cyberbullying incidents. A recent paper by Leung et al. (2018c), using a simulated Facebook setting manipulated the environment of a simulated Facebook setting into two conditions: the offending condition, in which comments that “support the cyber-bullies to further offend the cyber-victims” were shown vs. the defend condition, in which comments that “defend or help the cyber-victims” were shown among Hong Kong Chinese students. Results showed that only the defend condition promoted higher normative beliefs for cyber-bystanders to help the victims. Therefore, when educators promote intervening/defending behaviors of cyber-bystanders in the future intervention programs against cyberbullying, it is important for these programs to include perspectives of significant others of students. For instance, if cyber-bystanders believe that their peers, family members, etc., approve them to engage as “cyber-upstanders” to help cyber-victims, they are more likely to adhere to this subjective norm, which may help cyber-bystanders to become “upstanders” upon witnessing cyberbullying.

Another step of the five-step intervention model involves enhancement in self-efficacy to intervene. Self-efficacy is a self-related belief that has been widely studied in different psychological and educational studies. According to Bandura (1977), having high self-efficacy in a certain domain helps individuals to approach a situation in a more prosocial and confident manner, and several intervention programs on cyberbullying aimed at raising self-efficacy of students in combating cyberbullying (e.g., a recent one by Leung et al., 2019 has raised self- efficacy of college students to combat cyberbullying with a six-session constructivist-based anti-cyberbullying e-course). Consistent with previous findings, self-rated self-efficacy of students to intervene or stop cyberbullying predicted their intervening behavior (e.g., DeSmet et al., 2016); results of this study found that self-efficacy to intervene cyberbullying positively explained intention to cyberbullying, which, in turn, positively explained intervening behavior. Other recent studies in the Western context have also found that higher self-efficacy predicted a higher level of defending behavior among young Australian (e.g., Clark and Bussey, 2020) adolescents. Therefore, it seems that, regardless of the age and cultural background of students, it is important to target increasing the necessary defending and empathic skills for cyber-bystanders to intervene cyberbullying.

Besides the aforementioned characteristics of cyber-bystanders, results showed that past experience in cyberbullying victimization positively and directly explained intervening behavior; in other words, the participants who were cyber-victimized more in the past were more likely to demonstrate intervening behavior. However, past experience in cyberbullying perpetration (i.e., being cyber-bullies) did not explain intervening behavior. Findings from the current study are also in line with other past studies (e.g., Bussey et al., 2020; Clark and Bussey, 2020) on a younger population; they found that only past cyberbullying victimization, but not cyberbullying perpetration, was positively related to cyber-defending behavior. These results could be explained by other past studies, which suggested that past experience in being cyber-victimized may activate greater empathy, and empathy has been found to be a strong predictor of defending behavior (Van Cleemput et al., 2014).

Attitudes toward cyberbullying, PBC, and felt responsibility to intervene cyberbullying did not significantly predict intention to intervene nor intervening behavior in the path analysis model; therefore, only part of hypothesis 1 was supported. Ajzen and Fishbein (1977) proposed the “correspondence hypothesis,” which stated that, when attitudes and behaviors were measured at corresponding levels of specificity, the correlation between attitudes and behaviors would be higher. In other words, general attitudes predicted general behavioral tendencies, but only specific attitudes predicted specific behavior. In the present study, “intervening behavior” was a specific behavior to help the cyber-victims by intervening a cyberbullying circumstance; however, a scale that measured a more general attitude toward cyberbullying, instead of the specific attitude toward “intervening cyberbullying” was used. This low correspondence between attitude and behavior may explain the lack of significant relations among attitudes, intention to intervene, and intervening behavior in this study.

PBC means the level of confidence individuals have in their abilities to correctly perform a behavior (Ajzen, 1991). Although past studies found that PBC was one of the predictors of cyberbullying behavior (e.g., Heirman and Walrave, 2012), there is a lack of studies examining the role of PBC among cyber-bystanders. The current study found that PBC did not explain intention to intervene nor intervening behavior with regard to cyberbullying. Some past studies suggested that PBC is the weakest or non-significant predictor of behavior in the presence of other predictors (e.g., Greaves et al., 2013; Tipton, 2014; Prapavessis et al., 2015). Riemenschneider et al. (2011) also found that PBC did not significantly predict the behavior of students related to ethical decisions. As ethical judgments could vary by culture or other contextual factors, these contextual factors may, in turn, potentially affect the relationship between PBC and the behaviors of cyber-bystanders.

Felt responsibility did not explain intention to intervene nor intervening behavior in the current study, albeit felt responsibility has long been considered as an important factor to predict intervening behavior. A possible explanation is that felt responsibility can be perceived differently by students in the online context, in which other factors, such as anonymity and increased emotional distance between a victim and a bystander, may contribute to a weaker linkage between felt responsibility and intervening behavior. Concerning felt responsibility, as suggested by the study of Gahagan et al. (2016), college student participants reported that felt responsibility upon witnessing cyberbullying depends on circumstances, namely, “(a) personal connection to the cyber-victim, (b) personal morals regarding cyberbullying, and (c) personal capabilities to helping the cyber-victim” (p. 1103). These circumstantial factors, however, were not included in the current study when measuring felt responsibility. Future studies should consider examining the effects of circumstantial factors on intention to intervene and intervening behaviors.

As a cross-sectional design was employed in this study, therefore, the causal relation between measured variables cannot be drawn. Nevertheless, the results of the current study provide guidance and grounds for future investigations on causal or longitudinal relations among characteristics of cyber-bystanders and their intervening intention or behavior upon witnessing cyberbullying. Moreover, given the high prevalence rates of cyberbullying among undergraduate students (e.g., Dilmaç, 2009; Minor et al., 2013; Faucher et al., 2014), therefore, with an aim to fill up the gap in the existing literature on understanding behavior of cyber-bystanders in this under-researched population, the current study mainly examined the cyberbullying phenomenon among Chinese college students, who are “emerging adults” or adolescents older than typical teenagers. Our findings may not fully generalize to explain cyberbullying intervention in younger adolescents. Future studies can involve students from a wider age range to examine the developmental impacts on behaviors of cyber-bystanders. While only age and gender were collected and included as covariates in the current study, future studies can investigate the role of other sociodemographic variables (such as socioeconomic status, life events, and family relationships) in intervening behaviors of cyber-bystanders.

The current study only focused on psychosocial and cognitive characteristics in explaining cyberbullying intervention. It is possible that the focus of this study may overlook the effects of other predictive factors on intervening behaviors. Also, although gender and age were already included in the model as covariates, the gender ratio in the present study was imbalanced (with 76.9% of the female participants). However, as the study was conducted in Hong Kong, China, according to statistics (University Grants Committee, 2019) on the student ratio of female to male college students in Hong Kong, China, there are more female than male students in most universities (a female-to-male student ratio ranging from 1.13:1 to 3.17:1). Despite its limitations, findings from this study provide implications for future studies to further investigate the mechanism of socio-cognitive factors that explain the intention of cyber-bystanders to intervene and intervening in behavior upon witnessing cyberbullying in a Chinese population. As cyberbullied victims may suffer from a number of negative outcomes such as having more depressive symptoms and other socio-emotional problems (e.g., Olenik-Shemesh et al., 2012; Tennant et al., 2015), and that mood disorders and psychological strains are related to more mental health problems such as suicidal thoughts and behaviors (e.g., Zhang et al., 2020), and suicide rates are high among college students (e.g., Lew et al., 2020); it is important to develop anti-cyberbullying programs to combat cyberbullying. For instance, a recent study has found that attachment to peers and parents buffered depression symptoms among Chinese youth (e.g., Lan and Wang, 2020). Therefore, future cyberbullying programs may consider fostering interpersonal support, together with strengthening specific psychosocial resources or factors (e.g., awareness and self-efficacy to intervening cyberbullying) that this study found to promote positive bystander behaviors and safe cyberspace.

## Data Availability Statement

The datasets generated for this study are available on request to the corresponding author.

## Ethics Statement

The studies involving human participants were reviewed and approved by Human Research Ethics Committee, The Education University of Hong Kong. The participants provided their written informed consent to participate in this study.

## Author Contributions

AL: conceptualization, data collection/analysis, and write-up.

## Conflict of Interest

The author declares that the research was conducted in the absence of any commercial or financial relationships that could be construed as a potential conflict of interest.
